# 574. Human Immunodeficiency Virus (HIV) Testing and Evidence of HIV among Real-World Long-Acting Pre-Exposure Prophylaxis (PrEP) Users in a United States Claims Database: Results from the PrEPFACTS Study

**DOI:** 10.1093/ofid/ofaf695.183

**Published:** 2026-01-11

**Authors:** Aimee A Metzner, Gabrielle F Herman, Catherine Nguyen, Raj Desai, Shana Walko, Dora Martinez, Sherry Shi, Leili Young-Xu, Maral DerSarkissian

**Affiliations:** ViiV Healthcare, Durham, North Carolina; ViiV Healthcare, Durham, North Carolina; Analysis Group, Inc., Los Angeles, California; Analysis Group, Inc., Los Angeles, California; ViiV Healthcare, Durham, North Carolina; ViiV Healthcare, Durham, North Carolina; Analysis Group, Canada, Quebec, Canada; Analysis Group, Canada, Quebec, Canada; Analysis Group, Inc., Los Angeles, California

## Abstract

**Background:**

APRETUDE (cabotegravir long-acting [CAB-LA]) was approved for human immunodeficiency virus (HIV) pre-exposure prophylaxis (PrEP) in December 2021. The PrEPFACTS study described real-world HIV testing patterns and evidence of HIV among CAB-LA users.

**Figure:**
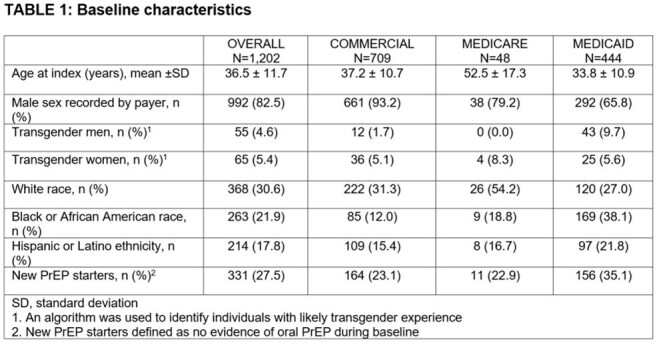

**Figure:**
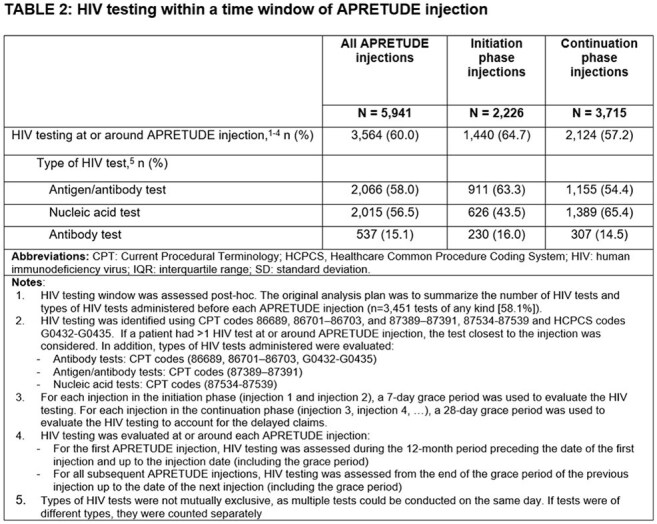

**Methods:**

PrEPFACTS was a retrospective US cohort study using data from the Komodo Research Database (01/Dec/2020-30/Sept/2023). Individuals ≥ 12 years old who received ≥ 1 CAB-LA injection (first injection defined as index date), had ≥ 12 months of continuous insurance eligibility before the index (i.e. baseline), and ≥ 6 months after were included. Individuals with HIV-1 or HIV-2 diagnosis or receiving ≥ 60 days of non-PrEP antiretroviral therapy (ART) during baseline were excluded. Individuals were followed from index to the earliest of either end of continuous enrollment, death, or end of data availability (i.e. follow-up). HIV testing patterns and first evidence of HIV (defined as an HIV diagnosis code and having oral ART on hand for ≥ 60 days) were assessed during follow up.

**Figure:**
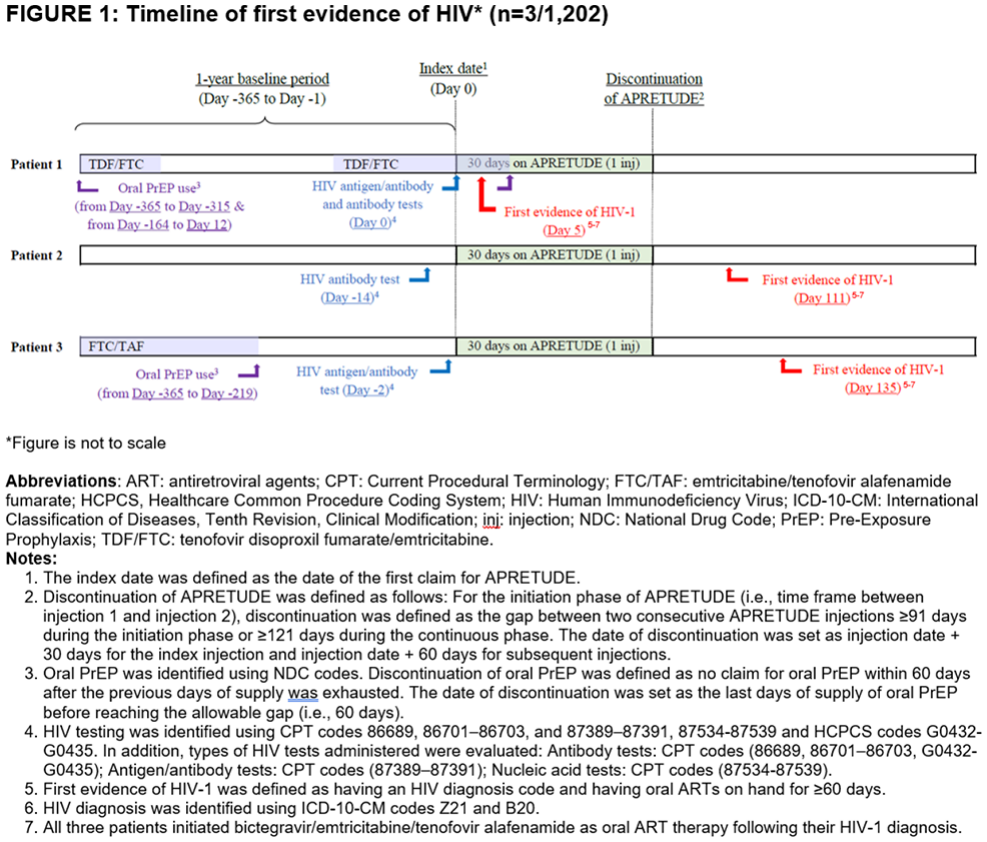

**Results:**

Among 1,202 eligible individuals (Table 1) with median follow up of 325 days (interquartile range [IQR] 242, 423), HIV testing occurred surrounding CAB-LA injections for 60.0% of all injections (n/N, 3,564/5,941; Table 2). When differentiated by initiation injections and continuation injections, HIV testing occurred for 64.7% (1,440/2,226) and 57.2% (2,124/3,715), respectively. Of the 1,202 individuals, three (0.2%) had evidence of HIV during follow up and initiated ART with bictegravir/emtricitabine/tenofovir alafenamide (Figure 1), while no evidence of HIV was observed for the remaining 99.8%.

**Conclusion:**

HIV testing observed in PrEPFACTS did not conform to CAB-LA product label or CDC guidelines, even though HIV testing is a key component of PrEP use. While the majority of injections had corresponding HIV tests, the database cannot capture tests that were not billed for and therefore some may be missing. PrEPFACTS also demonstrates from a large real-world sample that evidence of HIV after CAB-LA use is minimal with no evidence of seroconversions with on-time injections. For the few individuals with seroconversion, integrase inhibitor-based treatments were initiated after CAB-LA use, showing that first-line ART regimens were still utilized.

**Disclosures:**

All Authors: No reported disclosures

